# Influence of Physical Activity in Children and Adolescents with Cerebral Palsy: A Systematic Review

**DOI:** 10.3390/children12070853

**Published:** 2025-06-27

**Authors:** Faustino Andrés-Pérez, Lluna Maria Bru-Luna, Sergio Hidalgo-Fuentes, Fátima Llamas-Salguero, Manuel Martí-Vilar

**Affiliations:** 1 Department of Basic Psychology, Faculty of Psychology and Speech Therapy, Universitat de València, 46010 Valencia, Spain; fausanpe@alumni.uv.es (F.A.-P.); manuel.marti-vilar@uv.es (M.M.-V.); 2 Department of Education, Faculty of Social Sciences, Universidad Europea de Valencia, 46010 Valencia, Spain; lluna.bru@universidadeuropea.es; 3 Faculty of Education and Psychology, University of Extremadura, Av. de Elvas, S/n, 06006 Badajoz, Spain; fatimalls@unex.es

**Keywords:** physical activity, cerebral palsy, quality of life

## Abstract

Cerebral palsy (CP) is one of the most common disorders in childhood that significantly impacts quality of life. **Background/Objectives:** This study conducted a literature review of physical activity (PA)-based interventions for children and adolescents with CP, highlighting the physical, cognitive, and social benefits, as well as the factors and barriers that influence practice. **Methods:** The PRISMA methodology was used to identify and analyze the most relevant studies up to December 2024 through specific search equations and the databases Science Direct, Scopus, and Dialnet. Of the 707 articles identified, a total of 62 publications were selected for further analysis. These were subjected to a quality assessment through a checklist based on seven items. **Results:** The practice of PA guaranteed improvements in balance, postural control, strength, socialization, and self-confidence. On the other hand, some programs, such as Makey Makey, Ballet, aquatic PA, and Matrogymnasia, among others, were highlighted because they promoted an increase in physical practice among the population. However, there are still some barriers, such as sedentary lifestyles (90%), limited accessibility, and a lack of qualified professionals that hinder the intervention and ensure motivation and interest in its practice for those individuals who have not yet begun to practice it. **Conclusions:** PA is an effective tool to favor quality of life in children and adolescents, as it contributes to their integral development and social inclusion. The need for interdisciplinary strategies to reduce barriers and increase the benefits of PA is emphasized. A joint effort to promote integration in leisure time could promote optimal long-term results.

## 1. Introduction

Cerebral palsy (CP) is a persistent disorder characterized by limitations in locomotion and posture, affecting muscle tone, voluntary and involuntary movements, coordination, and balance; these barriers occur as a consequence of childhood brain injury [1,2,3,4]. The main cause related to such pathology is associated with alterations to the central nervous system (CNS) during the fetal development period [5] or brain injuries occurring in the prenatal and perinatal stages of the individual [6,7]. In developed countries, the prevalence of CP is estimated at 1.6 per 1000 births [8]. In Europe, the Surveillance of Cerebral Palsy in Europe [9] identifies a prevalence of two to three cases per 1000 newborns.

The symptoms associated with CP include a wide range of difficulties derived from alterations in the development of the central nervous system, which affect not only motor control but also muscle tone, coordination, and balance. Among the most common problems are motor dyscontrol, muscle weakness, and postural, motor, and psychomotor disorders, both fine and gross [10]. Taylor et al. [11] demonstrated that these deficiencies lead to a reduction in aerobic capacity and muscle strength, essential components of general health. These traits have significant implications for quality of life, as physical limitations often give rise as a consequence of other impairments in areas such as communication, perception, and behavior [12,13]. Each of these impairments, often accompanied by other sensory, cognitive, communicative, perceptual, and behavioral impairments or epileptic seizures, generate significant challenges in personal, social, family, and school adaptation, influenced by contextual factors such as family, educational, social, and health services [14].

Children with CP face multiple difficulties associated with their pathology, physical, cognitive, and social. The scientific literature identifies, through various investigations, problems related to deficits in language skills [15], executive functions, working memory [16], divided attention [17], response inhibition, and manipulative skills [18]. These adversities, combined with other documented social and behavioral barriers, significantly affect patients’ quality of life, becoming considerably reduced compared to the impact of other neurological disorders [19].

According to the classification criteria, CP is organized according to the type of motor involvement, anatomical distribution, and muscle tone. The main clinical forms include the following: spastic, dyskinetic, ataxic, and mixed, with spastic being the most prevalent, accounting for approximately 80% of cases [20]. Anatomical distribution defines other subtypes, such as tetraparesis, quadriparesis, hemiparesis, or monoparesis [21], while muscle tone can vary from isotonic to hypertonic or hypotonic. The Gross Motor Function Classification System [22] allows the assessment of functional levels in five categories, from walking without limitations to severely restricted mobility, thus facilitating a more personalized therapeutic approach [23].

Within this field, physical activity (PA) emerges as an essential tool for improving the quality of life of children and young people with CP. Verschuren et al. [13] highlighted that adapted exercise programs not only improve cardiovascular function and muscle strength but also reduce sedentary levels. Lauruschkus et al. [24] emphasized that the continued practice of physical exercise allows for the regulating and promoting of social integration and emotional well-being. In this vein, the World Health Organization (WHO) has recommended adapted physical activities at least three times a week because these interventions not only offer physical benefits but also promote inclusion and emotional development [25].

The scientific literature based on the research conducted justifies the benefits provided via PA in people with CP by strengthening their physical and functional capacities. Several intervention programs using PA have demonstrated improvements at various levels: increased aerobic capacity [24,26,27,28,29], increased strength in the upper and lower extremities [11,29,30,31], and improved control over the body and posture, focusing on body stability [31,32] and expanded movement possibilities [30].

The development of innovative programs that integrate technologies, such as virtual reality devices and exoskeletons, promises to transform the physical treatment landscape for children with CP. Authors such as Martinez-Gomez et al. [33] suggest that these tools can increase motivation and participation, while others, such as adapted games and group activities, improve adherence to exercise programs. Thus, the need to explore interdisciplinary approaches and emerging technologies to maximize the therapeutic benefits of PA in this population is underscored. Appropriate and effective physical exercise practice among people with CP, focusing on improving motor behaviors, wheelchair agility, daily living task skills, etc., acts advantageously on the functionality and physical condition of this population [34].

During physical practice, the use of assistive tools has contributed to the improvement of motor skills in children with CP. The implementation of assistive devices promotes the execution of functional movements, adjusting the supports to the individual needs presented by the individual and optimizing the intervention and therapeutic results. In addition, it is worth mentioning the contribution of virtual reality, already mentioned above, by contributing to the construction of a safe and controlled space to develop motor skills and favoring the child’s interest and commitment to the practice [33]. Satonaka and Suzuki [34] emphasize the key role of PA in the development of specific exercises that enhance the development of individual agility and functionality for the better management of the assistive supports they have, such as wheelchairs. These authors indicate that specific practices such as agility circuits, combined with moderate aerobic training, have a significant impact on endurance capacity and independence in daily activities. In addition, this type of activity helps prevent the appearance of possible secondary complications: muscular atrophy, decreased cardiorespiratory capacity, etc.

Moreover, the multidisciplinary approach makes a key contribution to the proper development of interventions. The collaborative work of physiotherapists, PA professionals, health technology engineers, and psychologists allows the development of more effective and personalized intervention programs. These actions guarantee improvements not only at the physical level but also at the emotional and social levels, seeking to ensure an improvement in the overall quality of life of the child with CP [33].

Along these lines, Satonaka & Suzuki [34] argue that delivering these interventions in a group and in an inclusive manner fosters greater adherence to long-term benefits. Therefore, they stress the importance of promoting PA from an inclusive point of view, ensuring that participants are equally enriched through the benefits of physical practice on personal development.

However, there are significant barriers to participation in these programs, including architectural barriers, a lack of adapted equipment, and families’ lack of awareness of the benefits of PA, and a shortage of trained professionals hinders the implementation of effective programs. However, factors such as family support and access to adapted sports facilities act as key facilitators [35].

The aim of this research was to review the scientific literature published to date on interventions and empirical research related to PA in children and adolescents with CP. This study sought to consolidate current knowledge, identify gaps in the literature, and propose future lines of research both within and outside this field. With regard to the specific objectives, the following should be emphasized: first, to identify the most recent interventions and empirical studies that have been developed in recent years and that employ physical practice as an intervention strategy in children and adolescents with CP; second, to analyze the benefits that PA can contribute to physical, cognitive, emotional, and social development in this population, with the aim of understanding its integral impact on the quality of life of this population; and finally, to examine the determining factors that influence the practice of physical exercise in this group, such as contextual barriers, social support, accessibility to resources, and motivational aspects, among others. The final product of this research aimed to provide the scientific field with a global, integrative, and updated vision of the importance of PA in the development and growth of children and adolescents with CP.

## 2. Materials and Methods

### 2.1. Design

This study conducted a literature review of the existing literature on this topic in order to achieve the proposed objectives. The search procedure was based on the variables PA and CP in the child and adolescent population and the relationship between them, which provide a positive connection and impact by providing benefits that reduce or prevent some of the negative consequences of the pathology in question.

The methodology used for the following literature review was the PRISMA model [36] (Annex I), which guarantees the application of the scientific method, as well as a better structuring of the search, screening, and selection of studies.

The process carried out for the effective development of this study was structured in several key stages. To begin with, a search for information was conducted in the Dialnet, Scopus, and ScienceDirect databases, using specific equations that ensured adequate collection. Subsequently, a filtering process was applied based on previously established inclusion and exclusion criteria, ensuring that only studies that met the imposed standards were collected for subsequent analysis. Next, a quality assessment was carried out using a checklist composed of five items, which made it possible to classify the selected studies according to their quality. Finally, the information collected was extracted, organized, and categorized into five sections to ensure a more detailed description. This structured approach ensured a comprehensive and efficient analysis of the data, adjusted to the objectives of the study.

### 2.2. Sources of Information

The present research directed its search through information sources such as Dialnet, Scopus, and ScienceDirect, seeking to find a wide range of studies on the subject in question, which allow a more globalized vision to be obtained, reaching publications belonging to different countries and languages. The search equation designed for the collection of studies was based on the two fundamental study variables, cerebral palsy (1) and physical activity (2), and the Boolean operator and was used for more effective compression and searching. The result was “Cerebral palsy AND physical activity”.

For greater clarity of the procedure to be followed during the selection of articles, the PICOS strategy [37] was used to formulate the question on which the review is based: What effects does physical activity (I) have as an intervention in the physical, cognitive, emotional and social development (O) of children and adolescents with cerebral palsy (P)? In its construction, the variable “C”, referring to the “comparative function”, is suppressed because there is not going to be a comparison with other types of populations or study variables. The PICOS strategy constituted the initial basis for the scientific literature search and selection process.

Table 1 below shows each of the search equations, together with the source of information chosen and the number of results obtained. The search process was carried out during the second half of May 2025.

### 2.3. Selection Criteria

This review established inclusion and exclusion criteria in order to guarantee the relevance and quality of the studies included. The criteria applied are detailed below.

1. The inclusion criteria contemplated empirical studies or interventions focused on PA, published in any language, whose sample is composed of persons with CP between the ages of 0 and 20 years. Also included were studies that analyzed factors that influence the practice of PA, as well as those that presented methodologies or tools aimed at promoting PA and its benefits.
2. On the other hand, the exclusion criteria discarded those articles derived directly from the inclusion criteria, studies published after 2024, synthesis studies, those comparing people with CP to people without disabilities or other disabilities, reliability studies of research instruments, and those in which the age of the participants was not accurately specified.

## 3. Results

### 3.1. Selection of Studies

**Figure 1:**
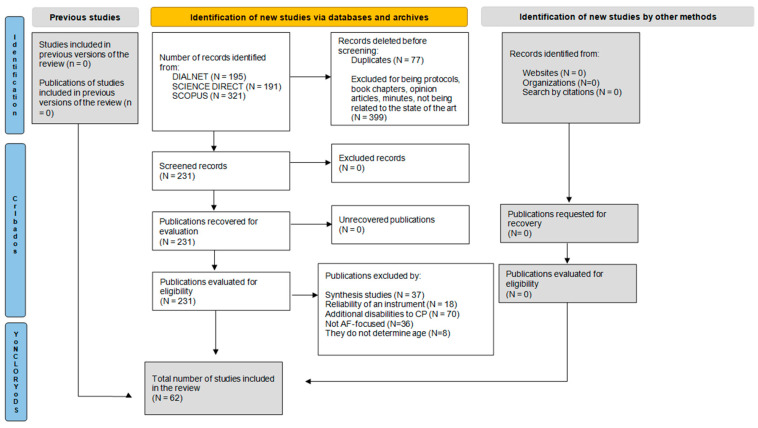
Flow chart according to PRISMA.

After the search process in the different databases, a total of 707 articles were identified, of which 77 were eliminated because they were duplicated in more than one database. Next, 399 articles were withdrawn after reading the title and abstract in the first screening process or because they were protocols, book chapters, opinion articles, or proceedings. Secondly, the remaining 231 articles were screened after the full body was read and the eligibility criteria were applied, resulting in a total of 62 publications. This selection and evaluation process was carried out rigorously and systematically by all the authors. After the selection process described above, the flowchart summarizing these steps is shown in Figure 1.

### 3.2. Selection Process

After the filtering process was completed, taking into account the established inclusion and exclusion criteria and following the PRISMA methodology, a quality assessment of the 62 articles selected for the study was carried out. The construction of the checklist for the quality analysis of the articles was carried out based on 75 criteria, using a model similar to that presented in other systematic reviews [38,39]. For an adequate assessment of the quality level, five items were established that evaluate the following: the description of the article (I.1); the sample (I.2), methodology (I.3), and results obtained (I.4); limitations (I.5) and a description on the duration, intensity, and practical modality of the intervention (I.6); and, finally, the effect size and confidence interval (I.7).

Together with three researchers with experience in pedagogical models and with extensive training in the field of psychology, a scale from 0 to 2 was established as an evaluation criterion, where 0 means not described, 1 means poorly described, and 2 means adequately described. In relation to this criterion, for the classification into categories of the studies according to the level of quality, three groups were established with a scale from 1 to 14 filled in based on the score of each article according to the seven items proposed at the beginning: low quality (0–4), medium quality (5–9), and high quality (10–14). The detailed scoring criteria and classification of studies are presented in Table 2.

### 3.3. Characteristics of the Studies

Table 3, presented below, provides a detailed description of the most relevant characteristics of each of the studies included in this systematic review. It summarizes key information such as the authors and year of publication, the objectives and duration of each study, the type and research design, the characteristics of the sample, and the main results and limitations, as well as the effect size and confidence interval.

### 3.4. Summary of the Studies

The selected articles corresponded to three main themes. On the one hand was research that examined the benefits of PA in children and adolescents with CP (*n* = 14); on the other hand were publications that analyzed the role of PA in the integral development of the individual with CP (*n* = 23), and finally, studies identified the factors that influence the practice of PA in this population (*n* = 23).

With the age of the sample taken as a classification criterion, there was a greater prevalence of articles that included children (*n* = 41) compared to studies that included adolescents (*n* = 4) and studies that included both (*n* = 18). On the other hand, according to the type of methodology used in the development of the research, a greater number of publications were found to follow a mixed methodology (*n* = 54), followed by publications of a qualitative (*n* = 8) and quantitative (*n* = 1) typology.

With reference to the quality criteria of the articles, a greater prevalence of high-quality publications can be observed (*n* = 37) compared to those classified as medium-quality (*n* = 25). Regarding the duration of the interventions, there was a higher percentage of interventions lasting from 1 month to 6 months (*n* = 19), followed by those lasting 1 week or less (*n* = 15). The studies that exceeded 6 months did not show much prevalence (*n* = 13).

According to the functionality criteria established by the GMFCS, we found a greater predominance of articles on interventions aimed at individuals at levels I, II, III, IV, and V (*n* = 10), followed by publications on levels I, II, and III (*n* = 9). Other research focused on more specific levels was also found, with less predominance than I and II (*n* = 7), II to IV (*n* = 3), I to IV (*n* = 2), and I (*n* = 1).

### 3.5. Benefits of PA Practice

In general, much research reflected the importance of regular and leisure time PA for children with CP to enhance, among others, motor skills [67], self-confidence and satisfaction, LPTA practice [53], quality of life [69], emotional intelligence [41] and a reduction in pain [56] and risk factors that are related to mental health disorders [50] caused by the multifactorial alterations that result from this pathology.

According to the study conducted by Keawutan et al. [68] with ambulatory and non-ambulatory CP children aged 4 to 5 years, it is important to create interventions and programs that promote PA in this population since they are people with a high rate of sedentary lifestyles and their daily lives are composed of a large percentage of inactive time. In this line, Rezavandzayeri et al. [41] demonstrated that physical exercise had a positive impact on the infant–juvenile population by improving their physical and psychological health, thus favoring their quality of life and emotional intelligence.

This positive impact was also advocated for by Гpигyc & Haгopнa [47], who showed that active participation in PA favored the construction of a more favorable body–function–structure and context ensuring better development, physical well-being, and motor control. In this direction, Keawutan et al. [69] reflected that gross motor skills and capacity were positively influenced by regular PA practice, and in turn, this gross psychomotor functionality was shown to be a factor that encourages individuals’ active participation in physical and pleasurable activities [40,68]. It could be considered as a continuous cycle in which each component is mutually reinforcing, promoting physical exercise, integral development, and mental health of the individual.

On the other hand, Ostergaard et al. [56] observed that children with CP presented certain pains in their body extremities that affect the performance of leisure PA. Therefore, these authors defended the importance of performing physical exercise since it helped reduce these discomforts and, in addition, produced an increase in motivation to practice leisure PA. However, despite the benefits of performing exercise, it was considered a great challenge for this population group since, as indicated by Gerber et al. [62], 90% of children with CP were sedentary. This situation led to an increased body fat percentage in this population, often reaching high levels, something that could be solved with the incorporation of this type of intervention in their daily routines [57]. From this approach, Rodrigues De Sousa et al. [40] stressed the need for these practices within this population to reduce sedentary lifestyles and raise awareness of the importance of performing PA in the future through pleasurable participation, enjoyment, and socialization.

### 3.6. Interventions in AF

On the other hand, the use of effective interventions allows children with CP to begin to enjoy and include PA within their daily routines and leisure time, ensuring a more continuous and increasing practice, which maintains the associated benefits. The results of this review have identified different methodologies. The “makey makey” approach allows for motivating and improving motor skills through a circuit board, which converts the individual’s physical contact into a digital signal that a computer interprets as a keyboard message, allowing receiving information from their movements [86]. Matrogymnastics, for its part, is considered an effective rehabilitation technique, as its exercises allow the child to develop his or her motor skills. These sessions are characterized by being carried out jointly with parents so that the exercises practiced can also be carried out in their homes [61].

Another of the methodologies used was the M2M musical movement, based on telecoaching training in which, through the viewing of videos, it is possible to increase PA during children’s free time [53]. On the other hand, Bania et al. [77] showed how training based on resistance to a force in an individualized way helps to improve muscle strength. However, in this same study, it was seen that this type of training did not motivate the practice of PA in leisure time, despite the benefits that it originates. The author argued that other methodologies and tools are needed to promote the practice of PA. The feasibility of using specific tools and instruments should also be highlighted. Hans and Fernández [73] used ankle–foot postural insoles in their study, which showed how the participant improved his balance capacity, reducing the anteroposterior and mediolateral sway that hindered his gait.

The results of this review have also identified the use of hippotherapy in the treatment of CP. Its importance in rehabilitation is based on three principles: heat transmitted from one organism to another, rhythmic impulses, and locomotion similar to human gait. These three factors activate various sensory and proprioceptive capabilities that reduce abnormal neurological behavior, promoting greater balance and postural control. In addition, a reduction in muscle spasticity was also observed producing benefits in the hip abductor muscles [74]. Paternina [90] also justified this positive impact in her study by developing gross motor skills and functional independence: the girl with whom the intervention was performed recovered the motor skills of the right arm, which she did not have before.

Another effective practice collected in this review was Participate CP, a therapeutic intervention that focused on participation as a means to achieve desired goals in PA performance. This tool is able to help reduce barriers that affect participation by addressing factors such as context, social relationships, environmental resources, skills, knowledge, and beliefs about one’s own abilities [64]. On the other hand, active video games that promote PA, such as dance or boxing, have also been used and have been shown to be beneficial tools promoting PA and, in turn, well-being [91]. Finally, progressive resistance training, conducted by Bar-Haim et al. [67] with 54 children aged 12 to 20 years showed improvements in habitual PA, social interaction, and motivation as a consequence of joint training.

Within the area of interventions, Ogonowska-Slodownik et al. [43] and Hamed et al. [49] highlighted the positive impact of the activities carried out in the aquatic environment since they improved performance, body control, balance, posture, and mobility. In addition, the authors highlighted these types of practices due to the motivational character and high degree of enjoyment generated among the participants during their execution. Similarly, another discipline that deserves special attention is that of Boccia [41], which guaranteed a wide range of benefits in quality of life by strengthening physical and psychological health and emotional intelligence, emphasizing its positive impact on perceptual, emotional, and critical competence, which are commonly compromised in individuals with CP.

### 3.7. Influencing Factors in the Practice of PA

Among the factors that can influence the performance of PA, the fundamental role of teachers and family members, as well as other people in the immediate environment, should be highlighted. The scientific literature has shown that environmental factors, the teacher’s attitude, and the materials used can positively or negatively affect the child’s growth and PA practice [70]. In their study, Figueiredo et al. [70] found that encouraging behaviors, positive social relationships, the availability of help, and adaptation favored participation; meanwhile, behaviors related to indifference, low responsiveness, exclusion, and overprotection hindered the practice of PA in adolescents with CP. Along these lines, Morris et al. [66] reported another series of facilitators that could help encourage physical practice in adolescents with CP: a sense of belonging, the role of the coach, security in continuing, and feeling supported.

Regarding other factors that may influence the performance of PA by children and adolescents with CP, Towns et al. [51] showed that people were afraid of losing their balance during PA because it could cause embarrassment and frustration but indicated that a pleasant environment could help in the execution of activities when confidence in balance is low. On the other hand, self-efficacy, age, and gross motor skills have been found to exhibit an association with moderate to vigorous PA [98]. Furthermore, Roth et al. [99], after analyzing lived experiences based on PA of 14 adolescents, saw that its performance helped children know themselves and communicate with others, as they considered it a way to connect and meet new people in the environment and feel emotions related to freedom despite the disability they present.

## 4. Discussion

The main objective of this research was to conduct a systematic review of the scientific literature published to date on PA interventions or empirical research on children and adolescents with CP in order to find out which ones exist, what benefits they produce in different areas of the person, and what factors influence the practice of PA in this population.

According to the WHO [100], the concept of health encompasses physical, mental, and social well-being. Several studies have argued that PA is a tool that acts beneficially on the population, improving health and cardiovascular capacity [101], decreasing the chances of suffering from cardiovascular problems [102], developing neuromuscular function [103], strengthening bone structure and functional capacity [100], and promoting gross motor skills and greater postural control [104] or proper gait [105]. These rehabilitation exercises also allow the child to adequately develop daily life tasks by gaining greater functional independence [106].

In relation to the first specific objective of identifying existing interventions or empirical research on PA in children and adolescents with CP, it should be noted that the literature reported less PA practice in this population than in those without disabilities [98,107]. Traditionally, different methodologies and tools have been investigated and applied for the rehabilitation and treatment of people with CP; however, none stand out as definitively the most effective in the literature [108,109]. Moreover, there is an insufficient study base to verify their use adequately [60,69,91]. While some of the methodologies identified through this systematic review, such as Makey Makey [86] or Music M2M [53], which encourage intrinsic and extrinsic motivation in relation to PA in the child and adolescent population with CP with the aim of benefiting from each of the advantages presented by its continuous practice, are worth highlighting.

When planning these interventions based on physical activity (PA), it is undoubtedly necessary to take into account the characteristics of the patient and the pathology presented [110]. Similarly, the sessions are not the same in adults and children. In adults with CP, PA periods last between 45 and 60 min since, for the benefits that originate from the performance of physical exercise to be significant, they must have a duration greater than 20 or 30 min [111]. In contrast, with children with CP, shorter sessions should be performed that are 15 or 20 min shorter than those with older children [110]. Following this approach, Wang et al. [46] emphasize the criterion of intensity during the development of physical practice, observing a more positive impact the higher the intensity of rehabilitation.

Regarding the second objective, focused on analyzing the benefits that PA can bring to physical, cognitive, emotional, and social development in this population, it has been seen that PA produces improvements in health in people with disabilities [112], physically [29,113], psychologically [114], and socially [115]. In addition, it has also been observed that PA practice causes greater levels of happiness in individuals, as it leads to increased well-being in young people [46,76]. On the other hand, the literature showed that people with CP usually present an affectation in the functional capacity of the upper limbs, which causes difficulties during the development of daily activities. These capacities could be improved with the execution of strategies or therapeutic interventions based on PA, which would give greater independence in the development of daily life tasks [106].

With regard to the last of the specific objectives, aimed at examining the determining factors that influence the practice of physical exercise in this group, a series of factors have been detected that can affect the development of PA. Researchers agree that one of the most common barriers that children with CP encounter when performing PA is laziness and not feeling comfortable with their body, together with recurrent thoughts of “I don’t feel capable” or “the technicians are not adequate” [13]. It is of great importance to know the barriers that people with disabilities present during physical exercise in order to be able to apply PA appropriately [116]. Another barrier encountered is pain [56] and the fear of losing balance [51]. In this sense, it is important that teachers create a space where the student feels comfortable and at ease to carry out physical practice without being afraid that he or she may make a mistake and be overcome by feelings of embarrassment and frustration. Here, it is worth highlighting the role of inclusive education since it can be a key element in the elimination of barriers that make it difficult for children and adolescents with CP to fully develop their abilities, promoting the construction of a space with equal opportunities and conditions [117,118,119]. In this sense, physical education would be a favorable tool for their development [119,120].

The studies analyzed in this review identified improvements in balance, muscle strength, postural control, etc. after interventions focused on PA in children and adolescents with CP. However, the clinical relevance is not clearly established. Prosser et al. [42] and Hamed et al. [49] identified improvements in gross motor and postural skills, but they did not specify whether the gains obtained exceeded the minimum clinically important difference (MCID), which partly limits the interpretation of the results obtained. On the other hand, Rezavandzayeri et al. [41] obtained positive results about the influence of physical practice on emotional intelligence and quality of life, but no relationships were established with their daily functionality around greater autonomy or independent mobility. Only a small percentage of the studies collected relate the achievements obtained to specific functional milestones such as independent sitting or ambulation, making it difficult to analyze how the benefits of PA also have a positive impact on the individual’s daily functioning. Based on this, we stress the need for future research to include data on MCID and establish relationships between the advances obtained in their abilities and their functionality in daily life, taking into account the levels of impairment established through the GMFCS.

On the other hand, for further research, it is also essential to develop interventions focused on PA practice using longitudinal designs that allow the evolution of the effects to be evaluated over longer periods of time. Likewise, it is essential to consider the degree of comorbidity present in individuals with the pathology since this can significantly influence their participation in PA. Including this variable in the analysis will contribute to a more comprehensive understanding of the factors that condition the results of the interventions and will provide a basis for designing more effective and personalized strategies.

### Limitations and Strengths

The limitations identified in this study can be grouped into different biases that affect its scope and reliability. Firstly, when analyzing the sample size used in the selected empirical publications, it was observed that some of them were characterized by being significantly reduced, interfering with the reliability of the results obtained and making it difficult to generalize them to larger populations.

Another barrier to be considered is the duration of the interventions studied. A wide range of the studies collected did not include a longitudinal follow-up to assess the sustainability of the effects once the intervention had ended. The lack of this analysis limits the ability to analyze the degree of permanence and effectiveness of the interventions in certain contexts. Another weakness of this review is related to its generalist approach since it offers a comprehensive view of the benefits and factors that influence the adequate practice of PA in the population of children and adolescents with CP but does not perform an exhaustive analysis of each of the variables identified. This global approach, despite presenting an effective understanding of the state of the question, presents this restriction to the particular effect of the variables. In this sense, we suggest the development of future research aimed at studying a specific factor, offering more precise evidence.

On the other hand, another limitation of this systematic review is related to the sources of information used. Data collection was based on the three databases: Dialnet, ScienceDirect, and Scopus, which provide relevant information in key areas such as education, psychology, and physical therapy. However, the results could have been enriched through the inclusion of other wide-ranging information sources, such as PubMed or Web of Science, which would have broadened the literary range of results. The integration of these sources would have strengthened the findings by providing a more comprehensive view of the topic addressed. On the other hand, the scarce inclusion of information collected directly from children with CP can be observed, with parents being the ones who provided the perspectives on how the intervention affected individuals’ development, excluding the key and subjective aspects of the experience for the person executing it. The veracity and robustness of the results could also be influenced by the lack of a bias assessment performed using the ROB 2 tool, which could add more veracity to the quality assessment checklist of the articles collected.

Finally, the need to broaden the range of information sources used in the review for greater information coverage is highlighted. Furthermore, in this specific case, the review focused on the child and adolescent population with CP, which limits the information selected to a specific group of individuals. Future research could focus on the effects of these interventions in the adult population, allowing a comparative analysis of the results.

Despite the aforementioned limitations, this study presents several strengths that support its validity and relevance. Firstly, the information covers the period from the first studies carried out in this population to the present day, which allows an analysis of how the different investigations and interventions have evolved. On the other hand, the review has included articles published in any language, thus avoiding linguistic bias and, in this way, avoiding offering a partial or distorted image of the literature.

Finally, a notable strength of this study, based on the quality assessment checklist of the articles previously presented (Table 2), identifies a high percentage of selected articles with high quality (72.5%), with only 17.5% corresponding to publications of medium quality. This result supports the relevance, solidity, and rigor of the information used in this review.

## 5. Conclusions

This systematic review gathered different studies on the influence of PA in people aged 0 to 20 years with CP, emphasizing not only the results of intervention focused on PA but also on possible methods that can be used to promote physical exercise in this population since it has been seen that people with CP, in general, present low levels of participation due to different barriers or limitations.

The low participation of this population in activities with motor and physical involvement is reducing their quality of life, which can further aggravate the limitations caused by their disability. Children and adolescents with CP are more likely to have greater body mass indexes and sedentary lifestyles, and although some have fat levels within the healthy range [57], it is important to promote awareness of the importance of introducing physical activity in leisure time (LPTA) into their lives to strengthen their quality of life at the physical, mental, personal, intellectual, and social levels.

The results of the literature review showed that PA has a beneficial effect on the vital development of these individuals. However, in several studies, one of the most common limitations reported by the researchers was the existence of a gap in knowledge on the subject due to the low percentage of interventions that exist so that awareness and sensitization efforts are essential in this regard.

Likewise, as theoretical implications, there is a need to expand the number of interventions that provide activities and techniques to be carried out with children and adolescents, allowing them to benefit from their practice, encourage motivation, enjoy them, reduce barriers, and improve participation, among others. In addition, we must not forget the importance of promoting inclusive education with the objective that the work and activities carried out are done jointly by all the agents of the context closest to the person, allowing the elimination of limitations and barriers present in society.

As practical implications, it is important to highlight the importance of educating and teaching society. The information provided by several studies shows how social and family factors, as well as the attitudes and actions carried out by the different agents that form part of the person’s immediate environment, can help to increase participation and motivation in PA. As shown by Lauruschkus et al. [24], factors such as feeling attached, feeling capable, enjoying it, and being aware that it is good to allow children to be more active and participatory.

## 6. Patents

The authors declare that there are no patent applications or grants related to the results described in this manuscript.

## Figures and Tables

**Table 1:** Search equations.

Source	Search Equations	Results
Science Direct	“Cerebral palsy” and “physical activity”	191
Scopus	“cerebral palsy” and “physical activity”	286
Dialnet	“Parálisis cerebral” and “physical activity”	192
Scopus	“Desarrollo motor” and “Parálisis cerebral”	35
Dialnet	“Actividad física” and “parálisis cerebral”	3

**Table 2:** Article quality evaluation.

Study	I.1	I.2	I.3	I.4	I.5	I.6	I.7	Degree of Quality
Rodrigues De Sousa et al. (2024) [40]	2	2	2	2	2	2	2	HQ
Rezavandzayeri et al. (2024) [41]	2	2	1	2	2	2	0	HQ
Prosser et al. (2024) [42]	2	2	2	2	2	1	1	HQ
Ogonowska-Słodownik et al. (2024) [43]	2	2	1	2	1	1	0	MQ
Benito et al. (2024) [44]	2	2	1	2	0	2	0	MQ
Yilmaz et al. (2023) [45]	2	1	2	2	0	1	0	MQ
Wang et al. (2023) [46]	2	1	2	2	2	2	0	HQ
Гpигyc & Haгopнa (2023) [47]	2	2	1	2	1	1	0	MQ
Hulst et al. (2023) [48]	2	2	1	2	0	1	0	MQ
Hamed et al. (2023) [49]	2	1	2	2	2	2	1	HQ
Cribb et al. (2023) [50]	2	1	1	2	2	0	1	MQ
Towns et al. (2022) [51]	2	2	2	2	0	0	0	MQ
Lee et al. (2022) [52]	2	2	2	2	2	1	0	HQ
Lai et al. (2022) [53]	2	1	1	2	2	2	1	HQ
Arruda (2022) [54]	2	1	1	2	0	2	0	MQ
Reedman et al. (2021) [55]	2	1	2	2	1	2	1	HQ
Ostergaard et al. (2021) [56]	2	2	2	2	0	1	1	HQ
Degerstedt et al. (2021) [57]	2	1	2	2	0	1	1	MQ
Williams et al. (2020) [58]	2	2	2	2	0	1	0	MQ
Wentz et al. (2020) [59]	2	2	2	2	2	2	0	HQ
Smit et al. (2020) [60]	2	2	2	2	2	1	0	HQ
Jami-Vargas et al. (2020) [61]	2	1	2	2	2	1	0	HQ
Gerber et al. (2020) [62]	2	2	2	2	0	0	0	MQ
Bjornson et al. (2020) [63]	2	1	2	2	2	1	0	HQ
Reedman et al. (2019) [64]	2	2	2	2	2	2	1	HQ
Orlando et al. (2019) [65]	2	2	2	2	0	1	1	HQ
Morris et al. (2019) [66]	2	2	1	2	0	1	0	MQ
Bar-Haim et al. (2019) [67]	2	1	1	2	2	1	0	MQ
Keawutan et al. (2018) [68]	2	2	1	2	2	2	0	HQ
Keawutan et al. (2018) [69]	2	2	1	2	2	1	1	HQ
Figueiredo et al. (2018) [70]	2	1	2	2	2	1	0	HQ
Schasfoort et al. (2017) [71]	2	2	2	2	2	1	1	HQ
Keawutan et al. (2017) [72]	2	2	2	2	0	2	0	HQ
Latorre-García (2017) [7]	2	1	2	2	2	1	0	HQ
Hanh & Fernández (2017) [73]	2	1	1	2	0	1	0	MQ
Antón (2017) [74]	2	2	2	2	0	2	0	HQ
Oftedal et al. (2016) [75]	2	2	2	2	2	2	0	HQ
Maher et al. (2016) [76]	2	1	2	2	2	1	0	HQ
Bania et al. (2016) [77]	2	1	1	2	0	2	0	MQ
Aidar et al. (2016) [78]	2	2	2	2	2	2	0	HQ
Ryan et al. (2015) [79]	2	2	2	2	1	2	0	HQ
Mitchell et al. (2015) [80]	2	2	1	2	2	2	0	HQ
Mitchell et al. (2015) [81]	2	1	2	2	1	2	0	HQ
Lauruschkus et al. (2015) [24]	2	1	1	2	0	2	0	MQ
Balemans et al. (2015) [82]	2	1	2	2	0	2	0	MQ
Van Wely et al. (2014) [83]	2	2	2	2	2	2	0	HQ
Van Wely et al. (2014) [84]	2	1	1	2	0	2	0	MQ
Shkedy et al. (2014) [85]	2	1	1	2	2	2	0	HQ
Lin & Chang (2014) [86]	2	2	2	2	2	2	0	HQ
Bania et al. (2014) [87]	2	1	1	2	0	2	0	MQ
Tang et al. (2013) [88]	2	1	1	2	1	2	0	MQ
Song (2013) [89]	2	1	1	2	2	1	0	MQ
Paternina (2013) [90]	2	1	2	2	2	2	0	HQ
Howcroft et al. (2012) [91]	2	1	2	2	1	2	0	HQ
Sandlund et al. (2011) [92]	2	1	2	2	1	2	0	HQ
Van Wely et al. (2010) [93]	2	2	2	2	2	2	0	HQ
Maher et al. (2010) [94]	2	2	2	2	1	2	0	HQ
Palisano et al. (2007) [22]	2	2	1	2	2	1	0	HQ
Maltais et al. (2005) [95]	2	1	1	2	2	1	0	MQ
Maltais et al. (2005) [96]	2	2	1	2	0	1	0	MQ
Chad et al. (1999) [97]	2	1	2	2	0	2	0	MQ
Van den Berg-Emons et al. (1998) [29]	2	1	1	2	0	2	0	MQ

Note. HQ: high quality. MQ: medium quality.

**Table 3:** Summary of the articles included in the review.

Authors and Year of Publication	Objectives and Duration	Type and Design of Research	Sample	Results	Limitations	Effect Size/Confidence Interval
Rodrigues De Sousa et al. (2024) [40]	Examined parents’ perceptions following a sports intervention for children with CP. Duration: eight weeks.	Descriptive, qualitative study	Sample size: 15 children. Sex: male and female. Age: 6 to 12 years old. Type or grade of CP: GMFCS levels I and II. Intensity: 1 h per week. Type of PA: Group sports.	The intervention had a positive impact on the children, favoring their motor and functional skills, socialization, and participation in pleasurable activities. In addition, it reduced sedentary lifestyles and raised awareness of the importance of PA for the future.	The results pertain to parents’ immediate perception of their children’s participation. Of note is the absence of a long-term perception.	NR.
Rezavandzayeri et al. (2024) [41]	Analyzed the influence of a Boccia program on the emotional intelligence and quality of life of children with CP. Duration: NR.	Mixed sectional–correlational study	Sample size: 165 children. Sex: male and female. Age: 20 years old. Type or grade of CP: levels IV and V according to the GMFCS. Intensity: 2–6 h per week. Type of PA: Boccia.	Motor training positively impacted quality of life by improving physical and psychological health and emotional intelligence, with emotional perception being the most affected.	The differentiation of functionality was made solely on the basis of GMFCS levels, without delving into other relevant aspects of motor functioning or complementary dimensions.	The size was 0.744 to 0.889, considered a large effect. NR.
Prosser et al. (2024) [42]	Analyzed the effect of Intensive Mobility Training therapy on gross motor development in children with CP. Duration: twenty-four weeks.	Mixed Design: randomized controlled clinical trial	Sample size: 42 children. Sex: male and female. Age: 12 to 36 months. Type or degree of CP: levels I, II, III, and IV of GMFCS. Intensity: three sessions per week. Type of PA: iMOVE.	Individuals improved their gross motor skills, postural control, and participation in daily activities. Intensive therapy could be considered a more effective program than standard care for gross motor development.	The physicians in charge of performing the intervention were leaders in the area, which may make the comparison greater than in other centers.	The effect size was 1.3 points, with a 95% confidence interval.
Ogonowska-Słodownik et al. (2024) [43]	Analyzed the functionality and enjoyment of children with CP after participating in aquatic PA. Duration: NR.	Mixed	Sample size: 10 children. Sex: male and female Age: 9- and 10-year-old PC. Type or degree of CP: GMFCS levels II to IV. Intensity: NR. Type of PA: aquatic therapy.	The children who participated showed improvements in their level of performance, mental adaptation, and balance control in the water. In addition, their feelings of enjoyment and motivation increased.	The sample size was small and the study was conducted only with the population of a single center.	NR.
Benito et al. (2024) [44]	Evaluated the effect of an adapted ballet program on children with hemiplegic CP. Duration: nine months.	Longitudinal experimental study	Sample size: 15 children. Sex: NR. Age: 7 to 10 years old. Type or degree of CP: level I in GMFCS. Intensity: 2/3 sessions per week. Type of PA: Adapted ballet.	The intervention showed a positive impact on balance, body symmetry, and active participation. The program produced physical, emotional, and social benefits.	NR.	NR.
Yilmaz et al. (2023) [45]	Analyzed the influence of stabilization physical exercises on balance and proprioception. Duration: twelve months.	Mixed experimental design	Sample size: 20 children. Sex: male and female. Age: 4 to 12 years. Type or degree of CP: levels I and II on the GMFCS. Intensity: NR. Type of PA: core stability exercises.	The exercises had a positive impact on proprioception, balance, and trunk involvement. These improvements positively influence their postural control and quality of life.	NR.	NR.
Wang et al. (2023) [46]	Analyzed the efficacy of constraint-induced movement therapy (CIMT) in children with CP. Duration: thirty-six hours.	Mixed randomized trial	Sample size: 50 children. Sex: NR. Age: 4 to 12 years. Type or degree of CP: unilateral CP. Intensity: 2 sessions per week. Type of PA: movement therapy with restriction.	Following the program, improvements in children’s gross psychomotor skills were identified. Some individuals showed improved use of the affected arm at 8 weeks. The intensity of the intervention intervened positively in motor rehabilitation.	The sample size was small and the intervention lasted 8 weeks, so the long-term effects could not be observed.	NR.
Гpигyc & Haгopнa (2023) [47]	Evaluated the effectiveness of dance and movement therapy in children and adolescents in palliative care. Duration: two years.	Qualitative, descriptive	Sample size: 12 children. Sex: male and female. Age: 8 to 12 years. Type or grade of CP: levels IV and V of the GMFCS. Intensity: NR. Type of PA: dance.	Participation had a positive impact on individuals in three areas: participation in PA, context (more favorable for the child), and body structure–function (physical well-being and motor control).	The study was conducted at a single center and with a small sample size.	NR.
Hulst et al. (2023) [48]	Evaluated physical activity during the 24-hour daily cycle and PA guidelines in children with cerebral palsy. Duration: seven days.	Mixed NR	Sample size: 54 children. Sex: male and female Age: 3 to 12 years. Type or degree of CP: levels I, II, and III according to the GMFCS. Intensity: 744.2 min/day. Type of PA: NR.	Low rates related to sleep, PA, and sedentary lifestyles seek to promote a call for improving healthy lifestyles and reducing negative health issues.	NR.	NR.
Hamed et al. (2023) [49]	Analyzed the effectiveness of aquatic physical activity on the gross motor skills of children with CP. Duration: twelve weeks.	Mixed randomized, controlled trial	Sample size: 34 individuals. Sex: NR. Age: 3 to 5 years. Type or degree of CP: spastic CP. Intensity: three weekly sessions. Type of PA: “10 steps” therapy.	After the intervention, a positive impact was observed on the Gross Motor Function Measure scores: posture, balance, and mobility.	The intervention had a short duration (12 weeks). The continuity of the progress was not analyzed once the intervention was completed.	NR. Ninety-five percent confidence interval.
Cribb et al. (2023) [50]	To identify the associations between PA practice and mental health in children with CP. Duration: NR.	Quantitative, cross-sectional	Sample size: 458. Sex: NR. Age: 6 to 17 years. Type or degree of CP: NR. Intensity: NR. Type of PA: NR.	Individuals with CP are more likely to present mental health disorders, anxiety, depression, and conduct disorders. PA significantly reduces risk factors.	The information is collected through interviews conducted with parents and caregivers, so there may be biases in memory or perception.	NR. Ninety-five percent confidence interval.
Towns et al. (2022) [51]	Analyzed the balance confidence of children with CP. Duration: NR.	Descriptive, qualitative study	Sample size: eight children. Sex: male and female. Age: 9 to 17 years. Type or degree of CP: levels I and II of the GMFCS. Intensity: NR. Type of PA: NR.	Young people are concerned about losing their balance during PA. Loss of balance causes embarrassment and frustration, rather than fear. Social factors can make an environment pleasant when confidence in balance is low.	NR.	NR.
Lee et al. (2022) [52]	Analyzed the relationship between habitual PA and its relationship with the quality of life in children with CP. Duration: seven days.	Mixed	Sample size: 46 children. Sex: male and female. Age: 7.48 average age. Type or degree of CP: levels I, II, and III of the GMFCS. Intensity: NR. Type of PA: regular PA.	Energy expenditure can be used as an indicator to look at PA and quality of life in children with CP. In addition, it can be used as a biomarker of these two characteristics.	The small number of children and the specific characteristics make it impossible to generalize the results.	NR.
Lai et al. (2022) [53]	Analyzed the effectiveness of movement with music (M2M) with the aim of increasing physical activity in leisure time (LTPA). Duration: four weeks.	Mixed	Sample size: 58 children. Sex: male and female. Age: 10 to 19 years. Type or degree of CP: CP. Intensity: three sessions per week. Type of PA: M2M.	Efficacy in the inclusion of an M2M project as there is an increase in LTPA in children with CP.	The study used a small sample; the implementation would need to be analyzed in a larger trial.	They identify an effect size of 0.17. NR.
Arruda (2022) [54]	To evaluate the sedentary behavior and PA of children with CP and their relationship with body composition. Duration: seven days.	Mixed NR	Sample size: 53 children. Sex: male and female. Age: 2 to 10 years. Type or degree of CP: dyskinetic CP. Intensity: mild, moderate, and vigorous. Type of PA: regular PA.	Children with dyskinetic CP practiced more PA and were less sedentary than spastic children. Children with more severe CP had a delay in growth that led to less physical activity.	NR.	NR.
Reedman et al. (2021) [55]	Determined the predictors that improve the participation of children with CP in PA during their free time using Participate CP. Duration: eight weeks.	Mixed randomized, controlled trial with a waiting list	Sample size: 33 children. Sex: male and female. Age: 8 to 12 years. Type or degree of CP: CP. Intensity: 1 h per week. Type of PA: Leisure time physical activity participate in CP.	Children with intrinsic motivation from the beginning improved more. Those who met the treatment objectives achieved greater participation during PA.	Small sample.	NR. Ninety-five percent confidence interval.
Ostergaard et al. (2021) [56]	To identify the level of pain in children with CP and its relationship with participation in leisure PA. Duration: one year.	Mixed cross-sectional study	Sample size: 960 children. Sex: male and female. Age: 2 to 11 years. Type or degree of CP: levels I, II, III, IV, and V of the GMFCS. Intensity: NR. Type of PA: leisure PA.	A large percentage of children with CP have pain, which influences leisure PA. Interventions to reduce pain are important, as they help to reduce pain and favor the practice of PA.	NR.	NR. Ninety-five percent confidence interval.
Degerstedt et al. (2021) [57]	Analyzed the influence of sex, country, and functional factors on leisure PA practice in children with CP in Sweden. Duration: one year.	Mixed cross-sectional study	Sample size: 1935 children. Sex: male and female. Age: 6 to 18 years. Type or degree of CP: CP. Intensity: NR. Type of PA: NR.	Children born in Sweden were more likely to perform PA than those born outside Europe. Boys with CP performed more leisure PA than girls.	NR.	NR. Ninety-five percent confidence interval.
Williams et al. (2020) [58]	To describe the energy expenditure, body composition, and nutritional intake produced via PA in adolescents with PC. Duration: NR.	Mixed cross-sectional study	Sample size: 12 children. Sex: male and female. Age: 12 to 19 years. Type or degree of CP: children with CP at GMFCS levels II, III, IV, and V. Intensity: moderate to vigorous. Type of PA: adapted PA.	Adolescents with CP may have a high percentage of fat and high levels of sedentary and moderate to vigorous PA. It is important to monitor body fat percentage in adolescents with CP as it tends to be high, even though some have a level within their healthy range.	NR.	NR.
Wentz et al. (2020) [59]	Analyzed gait and PA in children with CP while considering gross motor function, age, and geographical location to assess the validity of the Early Activity Scale for Endurance. Duration: seven days.	Mixed prospective cohort study	Sample size: 79 children. Sex: male and female. Age: 11 years. Type or degree of CP: children with CP at levels I, II III, IV, and V of the GMFCS. Intensity: moderate to vigorous. Type of PA: regular PA.	Only the different levels of gross motor function marked significant differences in gait and moderate to vigorous PA.	The sample size, being so small, means that not as much data comes out as could exist.	NR.
Smit et al. (2020) [60]	Analyzed sleep and the relationship between its quality, PA, and sedentary behavior in children and adolescents with CP. Duration: seven days.	Mixed cross-sectional study	Sample size: 36 children. Sex: male and female. Age: 15 years. Type or degree of CP: children with spastic CP at levels I, II, III, and IV of the GMFCS. Intensity: NR. Type of PA: NR.	Children with CP have a recommended sleep duration and this is a factor that is related to the sedentary nature of children.	Little research based on different measures of sleep quality and quantity.	NR.
Jami-Vargas et al. (2020) [61]	To improve the development of gross motor skills in children with CP using the Matrogymnastics method. Duration: NR.	Mixed, descriptive	Sample size: 16 children. Sex: male and female. Age: 7 years. Type or degree of CP: PCI. Intensity: NR. Type of PA: matrogymnasia.	Matrogymnasia is an effective tool for the motor development of children, adding the participation of parents so that the exercise continues at home.	Longitudinal study, because after the study period, it is not clear whether the effects have been maintained or not.	NR.
Gerber et al. (2020) [62]	Analyzed the PA and gross motor skills of children and adolescents with CP and tried to understand their relationship. Duration: NR.	Qualitative clinical trial	Sample size: 25 children. Sex: male and female. Age: 8 to 20 years. Type or degree of CP: GMFCS level I, II, and III. Intensity: NR. Type of PA: NR.	The children spent 90% of their time sitting or passive and most of them had difficulty performing PA beyond 3 min. We did not see a strong relationship between motor ability and performance, but we did observe that a certain level of motor ability is necessary for PA performance.	There is no software that allows routine data analysis by healthcare providers who are not trained in clinical settings.	NR.
Bjornson et al. (2020) [63]	Analyzed the trajectories for the development of AF and walking function in children with CP. Duration: NR.	Mixed longitudinal study	Sample size: 79 children. Sex: male and female. Age: 3 to 12 years old. Type or degree of CP: children with CP of GMFCS levels I, II, III, IV, and V. Intensity: NR. Type of PA: marching.	The children did not reflect plateaus in PA or gait according to functional level; however, they showed a decrease in the quantity and quality of PA from 3 to 12 years of age.	NR.	NR.
Reedman et al. (2019) [64]	To determine the efficacy of a participation-based therapy to improve performance and satisfaction with free and habitual PA. Duration: twelve weeks.	Mixed	Sample size: 37 children. Sex: male and female. Age: 10 years old. Type or degree of CP: levels I, II, and III according to the GMFCS. Intensity: Light, moderate, and vigorous. Type of PA: Habitual PA.	Positive results were observed in increasing the performance of leisure PA goals by reducing barriers influencing participation.	The program did not produce changes in the average habitual PA.	NR. Ninety-five percent confidence interval.
Orlando et al. (2019) [65]	Analyzed self-initiated PA and its relationship with gross motor skills and the participation of children with CP. Duration: seven days.	Mixed NR	Sample size: 20 children. Sex: male and female. Age: 1–3 years. Type or degree of CP: non-ambulatory CP. Intensity: slight, moderate, and vigorous. Type of PA: NR.	The results showed non-significant values in the performance of games on the ground in gross motor skills and participation.	Independently measure PA, participation, and gross motor skills.	NR.
Morris et al. (2019) [66]	To determine the facilitators that promote PA in adolescents with CP. Duration: NR.	Mixed inductive, thematic study	Sample size: 15 children. Sex: male and female. Age: 12 to 18 years old. Type or degree of CP: children with CP at levels I, II, III, IV, and V according to the GMFCS. Intensity: NR. Type of PA: recreational.	They drew seven ideas: start, want to succeed, sense of belonging, the importance of the coach, endorsement to continue, support, and being passionate. These were synthesized into the Framework for Sustained Engagement.	NR.	NR.
Bar-Haim et al. (2019) [67]	Analyzed the changes in habitual PA and motor skills after the exercises. Duration: twelve weeks.	Mixed NR	Sample size: 54 children. Gender: male and female. Age: 12 to 20 years old. Type or degree of CP: bilateral spastic CP of GMFCS levels II and III. Intensity: Moderate to vigorous. Type of PA: Habitual PA.	Motor capacity improves after the interventions by seeing a growth in habitual PA. Intervention based on progressive group resistance training produces improvements in habitual PA, improving the social interaction and motivation that joint training brings.	NR.	NR.
Keawutan et al. (2018) [68]	Analyzed the relationship between habitual physical activity, sedentary behavior, and motor skills in children with CP. Duration: seven days.	Mixed cross-sectional study	Sample size: 67 children. Sex: male and female. Age: 4 to 5 years old. Type or degree of CP: PCI classified according to the GMFCS. Intensity: Light, moderate, and vigorous. Type of PA: Habitual PA.	Gross motor skills and ability are influenced by habitual PA and sedentary lifestyles.	Those children with progressive disorders were not included.	NR.
Keawutanet et al. (2018) [69]	Evaluated the quality of life in 5-year-old children with CP and related it to PA. Duration: seven days.	Mixed cross-sectional study	Sample size: 58 children. Sex: male and female. Age: 5 years. Type or degree of CP: levels I, II, III, IV, and V of the GMFCS. Intensity: Light, moderate, and vigorous. Type of PA: NR.	Children who engage in PA have a better quality of life than those who do not, due to the feelings of emotional well-being, self-esteem, and control of motor function that PA produces.	The habitual PA was not related to the quality of life reported by the parents.	NR. Ninety-five percent confidence interval.
Figueiredo et al. (2018) [70]	Analyzed the factors that influence the participation in PA among students with CP. Duration: NR.	Qualitative NR	Sample size: 10 people. Sex: male and female. Age: 12 to 14 years. Type or degree of CP: levels I to IV on the GMFCS. Intensity: NR. Type of PA: Team sports and recreational activities.	Personal and environmental factors, the attitudes of teachers, monitors, peers, and the variety of materials have a positive or negative influence on these 3 categories: “There is no way I can participate” (1), “I participate when” (2) and “It would be easier if” (3).	Unique perspective from the viewpoint of teenagers, without considering other agents in the environment.	NR.
Schasfoort et al. (2017) [71]	Evaluated the treatment with botulinum toxin and its combination with intensive physiotherapy. Duration: six months.	Mixed NR	Sample size: 65 children. Sex: male and female. Age: 4 to 12 years old. Type or degree of CP: CP. Intensity: NR. Type of PA: therapeutic and functional.	Combination treatment is not cost-effective when seeking to improve gross motor skills, PA levels, and quality of life.	NR.	NR. Ninety-five percent confidence interval. Intervalo de confianza del 95%.
Keawutan et al. (2017) [72]	Analyzed the PA of children aged 4 to 5 years with CP and sedentary behavior. Duration: seven days.	Mixed comparative analysis	Sample size: 7 children. Sex: male and female. Age: 4 to 5 years. Type or degree of CP: children with ambulatory and non-ambulatory CP. Intensity: light, moderate, and vigorous. Type of PA: NR.	Children spend a large part of their time in inactivity. Interventions are needed to reduce sedentary lifestyles and increase habitual PA.	Little basis of interventions to reduce sedentary behavior and promote PA.	NR.
Latorre-García (2017) [7]	Analyzed the relationship between water-based PA and motor development in children with CP. Duration: twelve weeks.	Mixed conceptual and experimental bibliography	Sample size: 12 children. Sex: male and female. Age: between 14 and 36 months. Type or degree of CP: CP. Intensity: two times per week. Moderate. Type of PA: Aquatic.	Infants with PCI were able to develop gross motor skills. They also strengthened others such as balance, coordination, motor learning, muscle tone, and endurance.	The presented study spans from September 2013 to December 2016. Although it continues to this day, no data is presented.	NR.
Hanh & Fernández (2017) [73]	Evaluated the effect of using postural insoles and ankle–foot orthoses on static and functional balance in children with CP. Duration: six weeks.	Mixed	Sample size: 20 children. Sex: male and female. Age: 4 to 12 years old. Type or degree of CP: spastic diplegic CP and levels I and II of the GMFCS. Intensity: NR. Type of PA: functional and balance.	The use of such supports improves balance and reduces anteroposterior and mediolateral sway.	NR.	NR.
Antón (2017) [74]	Analyzed whether hippotherapy produces long-term benefits in muscle spasticity. Duration: eight weeks.	Mixed NR	Sample size: 44 children. Sex: male and female. Age: 8–9 years Type or degree of CP: NR. Intensity: two per week. Type of PA: hippotherapy.	Children benefited from hip abductor strengthening following hippotherapy.	The intervention program lasts 12 weeks.	NR.
Oftedal et al. (2016) [75]	To describe the relationship between height, growth velocity, habitual PA, energy intake, and sedentary lifestyle. Duration: two years.	Mixed longitudinal study with mixed effects regression models	Sample size: 175 children. Sex: male and female. Age: 18 months to 5 years. Type or degree of CP: children with levels I, II, III, IV, and V according to the GMFCS. Intensity: Light, moderate, and vigorous. Type of PA: habitual PA.	Functional status and gestational age are two factors to take into account when assessing the growth of children. Increasing active time and PA is effective in improving growth and health.	NR.	NR.
Maher et al. (2016) [76]	Analyzed the relationships between PA and quality of life with the health and happiness of young people with CP. Duration: NR.	Mixed cross-sectional study	Sample size: 70 children. Sex: male and female. Age: 13 years and 11 months. Type or degree of CP: levels I, II, III, IV, and V children according to the GMFCS. Intensity: light, moderate, and vigorous. Type of PA: recreational and outdoor.	A positive relationship was found between PA, quality of life, and happiness. PA has potential benefits for improving the well-being of young people. PA is a predictor of children’s quality of life, health, and happiness.	Lack of clinical services and interventions aimed at increasing PA in children with CP.	NR.
Bania et al. (2016) [77]	Analyzed whether individualized resistance training increases the daily PA of children with CP. Duration: twelve weeks.	Mixed cross-sectional study	Sample size: 36 children. Sex: male and female. Age: 13 years and 11 months. Type or degree of CP: children with bilateral spastic CP. Intensity: three per week. Moderate and high. Type of PA: progressive resistance PA.	Resistance training can improve muscle strength, but it does not increase PA practice. Other strategies are needed to address low levels of PA.	Lack of strategies to address the low daily PA in young people with CP.	NR.
Aidar et al. (2016) [78]	Analyzed social function and aquatic PA in children with CP using the Pediatric Disability Assessment Inventory. Duration: twelve weeks.	Mixed NR	Sample size: 21 children. Sex: male and female. Age: 6 to 12 years. Type or degree of CP: CP. Intensity: 2–3 per week. Slight and moderate. Type of PA: aquatic.	The practice of aquatic exercises improved motor skills and, in a transversal way, social function, thus promoting the independence of the child with CP.	NR.	NR.
Ryan et al. (2015) [79]	To relate sedentary behavior, PA, and cardiorespiratory fitness in children with CP. Duration: seven days.	Mixed cross-sectional study	Sample size: 55 children. Sex: male and female. Age: 6 to 17 years. Type or degree of CP: Children with unilateral CP. Type of PA: habitual and spontaneous PA. Children with ambulatory CP of levels I and II of the GMFCS. Intensity: Light, moderate, and vigorous.	Vigorous activity is related to cardiorespiratory fitness in children with CP, but not light or moderate activity. Children with CP have low levels of cardiorespiratory fitness, central adiposity, and high blood pressure.	The cross-sectional design does not allow for establishing causal relationships.	NR.
Mitchell et al. (2015) [80]	Analyzed the PA and the proportion obtained by performing 60 min of moderate to vigorous PA daily in children and adolescents with CP. Duration: seven days.	Mixed cross-sectional study	Sample size: 102 children. Sex: male and female. Age: 11 years old. Type or degree of CP: children with unilateral CP of GMFCS levels I and II. Intensity: Light, moderate, and vigorous Type of PA: Habitual PA.	A high percentage of children with unilateral CP do not perform the recommended amount of PA to meet health recommendations.	Only children with unilateral CP were selected.	NR.
Mitchell et al. (2015) [81]	Analyzed the influence of physical, personal, and environmental characteristics on the practice of PA in children with unilateral CP. Duration: seven days.	Mixed cross-sectional study	Sample size: 102 children. Sex: male and female. Age: 8 to 17 years. Type or degree of CP: children with unilateral CP independent of GMFCS levels I and II. Intensity: Light, moderate, and vigorous. Type of PA: habitual and spontaneous PA.	Young age, gender, greater walking endurance, and greater participation in the home and community are factors that influence the PA of children with CP.	Only children with independent unilateral CP were selected.	NR.
Lauruschkus et al. (2015) [24]	Analyzed the experiences of children with CP, participation in PA, and the facilitators and barriers. Duration: twelve weeks.	Mixed Exploratory analysis study	Sample size: 16 children. Sex: male and female. Age: 8 to 11 years. Type or degree of CP: CP. Intensity: slight and moderate. Type of PA: individual.	Facilitators are summarized as: enjoying the feeling, being able, feeling connected, being aware that it is good for me, children want to be physically active, they want to have fun and enjoy the feeling of speed and doing new activities that encourage participation. Barriers are fatigue and accessibility to some activities or places.	The study could have been improved by asking the children about the physical activities they enjoy.	NR.
Balemans et al. (2015) [82]	Investigated the changes in physical fitness and PA levels related to walking and fatigue in children. Duration: one year.	Mixed analysis of a randomized, controlled trial	Sample size: 46 children. Sex: male and female. Age: 7 to 13 years. Type or degree of CP: children with bilateral and unilateral CP. Intensity: slight and moderate. Type of PA: walking.	In children with bilateral CP, there is a positive relationship between physical fitness and gait-related PA, whereas in unilateral CP, there was no connection.	The random coefficient regression analysis cannot be used to establish a causal relationship.	NR.
Van Wely et al. (2014) [83]	Analyzed the effectiveness of a 6- month PA program on social participation, self-perception, and quality of life. Duration: six months.	Mixed multicenter controlled trial with blinded allocation and evaluations	Sample size: 49 children. Sex: male and female. Age: 7 to 13 years. Type or degree of CP: children with spastic CP. Intensity: slight and moderate. Type of PA: habitual PA.	The intervention had positive benefits on social participation in domestic life at 12 months, not at 6 months. No relationship was found with social participation in recreation, leisure, self-perception, and quality of life.	NR.	NR.
Van Wely et al. (2014) [84]	Analyzed whether a 6-month PA program produces benefits in the child with CP. Duration: six months.	Mixed	Sample size: 49 children. Sex: male and female. Age: 7 to 13 years. Type or degree of CP: spastic CP and GMFCS severity I, II, and III. Intensity: mild and moderate. Type of PA: regular PA.	The program combining PA, counseling, and home therapy was not effective in children with CP. There was a trend of improvement in the children’s attitudes, clinically irrelevant, not for 6 months, but for 12 months.	Examined each variable of the population that intervenes separately to understand its influence.	NR.
Shkedy et al. (2014) [85]	Analyzed the duration and indicators of PA in the population in the Middle East. Duration: seven days.	Mixed NR	Sample size: 222 children Sex: male and female. Age: 16 years old. Type or degree of CP: children with bilateral CP of GMFCS levels II, III, and IV. Intensity: Light, moderate, and vigorous. Type of PA: regular PA.	The Mann–Whitney U test showed that there were differences in gross motor function and lower walking, standing, and sedentary activity at higher GMFCS levels.	Limited control over participant compliance may have resulted in some records being defective.	NR.
Lin & Chang (2014) [86]	Analyzed whether the Makey Makey program is useful for developing an intervention project. Duration: four weeks.	Mixed NR	Sample size: 1 girl. Sex: female. Age: 5 years and 9 months. Type or degree of CP: convulsive CP. Intensity: Three per week. Mild and moderate. Type of AF: Makey Makey.	Useful tools to motivate and improve children’s motor skills. Helen, a child who could not stretch her arm, improved her ability to extend it and pick up objects from a table with it. Each time she did so, she received a stimulus given by the program, which encouraged her intrinsic motivation.	This approach could have been used to receive interactive feedback independent of physical disabilities.	NR.
Bania et al. (2014) [87]	Analyzed daily PA levels in adolescents with CP and tried to show factors that help predict these levels. Duration: seven days.	Mixed. cross-sectional study	Sample size: 45 children. Sex: male and female. Age: 15 to 20 years. Type or degree of CP: children with bilateral spastic CP of GMFCS levels II and III. Intensity: light, moderate, and vigorous. Type of PA: habitual PA.	Adolescents and young adults with bilateral spastic CP who can walk with difficulty have reduced PA. Gross motor function is a predictor of daily PA.	The number of variables was limited by the sample and its size.	NR.
Tang et al. (2013) [88]	To evaluate the use of an activity monitor to observe PA in children with mobility problems due to CP. Duration: seven days.	Mixed evaluation study	Sample size: 15 children. Sex: male and female. Age: 5 to 17 years old. Type or degree of CP: children with ambulatory CP. Intensity: light, moderate, and vigorous. Type of PA: regular PA.	The activity monitor made it possible to analyze the sitting and upright postural states and the importance of taking into account the degree of severity according to the time and type of steps taken (walking, tiptoeing). PA in leisure time showed improvements.	NR.	NR.
Song (2013) [89]	Analyzed the relationship between physical, cognitive function, and daily PA in children with CP. Duration: NR.	Mixed NR	Sample size: 78 children. Sex: male and female. Age: 1 to 43 months. Type or degree of CP: CP. Intensity: NR. Type of PA: regular PA.	Physical and cognitive functions change whether the person can stand with assistance or without assistance. Age did not have a great influence. Cognitive function is related to physical function. Daily PA was affected by each type of CP.	Cognitive and physical impairments are not included in the examination and evaluation of CP.	NR.
Paternina (2013) [90]	Evaluated hippotherapy as a therapeutic alternative. Duration: twelve weeks.	Qualitative NR	Sample size: one person. Sex: female. Age: 4 years and 7 months. Type or degree of CP: dyskinetic CP. Intensity: Two per week. Mild and moderate. Type of PA: Hippotherapy.	Positive impact on the child’s development, mainly in gross motor skills and functional independence. The child developed skills in the right arm that she did not have before.	There is no assessment of basic devices that influence cognitive development such as visual acuity and hearing.	NR.
Howcroft et al. (2012) [91]	Analyzed the potential of active video games to promote PA and rehabilitation therapies in children with CP. Duration: eight weeks.	Mixed single-group experimental study	Sample size: 17 children. Sex: male and female. Age: 9 years. Type or degree of CP: CP. Intensity: Three per week. Thirty min. Moderate. Type of PA: dances and sports.	Moderate levels of PA were seen during dance and boxing practice. Angular velocities and accelerations were significant in the dominant arm. High levels of enjoyment. Positive tool to encourage light and moderate PA.	The small base of studies on variation in individual movements and playing styles.	NR.
Sandlund et al. (2011) [92]	Analyzed the practice of low-cost interactive games as a home-based intervention for children with CP. Duration: eight weeks.	Qualitative exploratory analysis	Sample size: 14 children. Gender: male and female. Age: 6 to 16 years old. Type or degree of CP: CP. Intensity: Two per week. Thirty–forty-five min. Light and moderate. Type of PA: Interactive movement games.	Motivation and practice compliance were high. PA increased during the intervention. Children’s physical performance improved.	More specific analysis of motor functions.	NR.
Van Wely et al. (2010) [93]	Analyzed the LEARN 2 MOVE 7–12 as a program to improve PA based on physical training and lifestyle. Duration: six weeks.	Mixed	Sample size: 50 children. Sex: male and female. Age: 7 to 12 years. Type or degree of CP: children with GMFCS levels I, II, and III spastic CP. Intensity: mild to moderate. Type of PA: habitual PA.	Lifestyle change and physiotherapeutic interventions improve physical training and PA in children with CP.	The effects obtained from the intervention cannot be related to a specific element.	NR.
Maher et al. (2010) [94]	Analyzed a PA-based intervention using the internet for 8 weeks in adolescents with CP. Duration: twelve weeks.	Mixed	Sample size: 72 children. Sex: male and female. Age: 12 to 16 years old. Type or degree of CP: unilateral and bilateral CP of levels I, II, and III of the GMFCS. Intensity: moderate. Type of PA: remote PA.	Short-term improvements in PA and knowledge were seen. Not comparable to face-to-face PA.	In order to present the results, it has focused on the best evidence.	NR.
Palisano et al. (2007) [22]	Analyzed PA from the point of view of adolescents with CP. Duration: NR.	Qualitative descriptive study	Sample size: 156 children. Sex: male and female. Age: 11 to 17 years old. Type or degree of CP: children with CP of GMFCS levels I, II, III, IV, and V. Intensity: slight and moderate. Type of PA: daily and recreational PA.	The performance of adolescents in PA with CP changed according to the degree of GMFCS. Depending on the level, there were greater difficulties in performing PA.	Lack of collaborative work between therapists and PA teachers working in clinical settings to carry out fitness programs.	NR.
Maltais et al. (2005) [95]	Analyzed the relationship between PA levels and oxygen costs during walking. Duration: NR.	Mixed NR	Sample size: 11 children. Sex: male and female. Age: 10 to 16 years old. Type or degree of CP: mild CP. Intensity: low and moderate. Type of PA: regular PA and walking.	Those with a low PA level had a higher oxygen cost when walking.	Lack of interventions to analyze whether interventions aimed at reducing the cost of oxygen improve the level of PA and vice versa.	NR.
Maltais et al. (2005) [96]	Analyzed the relationship between habitual PA and the biomechanical economy of treadmill walking. Duration: NR.	Mixed controlled trial	Sample size: 11 children. Sex: male and female. Age: 10 to 16 years. Type or degree of CP: mild CP. Intensity: NR. Type of PA: habitual PA and walking.	Those who possessed a high biomechanical gait economy on the treadmill were more physically active. Treadmill speed affected gait biomechanics, but not time.	NR.	NR.
Chad et al. (1999) [97]	Analyzed the effect of a weight-bearing PA program on bone content and bone density in children with CP. Duration: twenty-four weeks.	Mixed experimental study	Sample size: 18 children. Gender: male and female. Age: 9 years old. Type or degree of CP: spastic CP. Intensity: moderate and high. Type of PA: weight-bearing PA.	The intervention produced improvements in bone mineral accumulation. Minimal loading period positively affects patients osteogenically.	NR.	NR.
Van den Berg-Emons et al. (1998) [29]	Analyzed the influence of 9-month PA programs on the daily PA, fat, and physical fitness of children. Duration: twelve weeks.	Mixed controlled trial	Sample size: 20 children. Sex: male and female. Age: 9 years old. Type or degree of CP: spastic CP. Intensity: moderate and high. Type of PA: endurance, strength, and mobility training.	Aerobic training has a limited effect on PA in children with CP but slows deterioration and muscle strength. The training improves maximal aerobic power.	NR.	NR.

Note: cerebral palsy; CIMT: constraint-induced movement therapy; GMFCS: Gross Motor Function Rating System; LTPA: physical activity in leisure time; M2M: movement with music; NR: not reported; PA: physical activity.

## Data Availability

All the data supporting the findings of this study are contained within the manuscript.

## Abbreviations

The following abbreviations are used in this manuscript:

ASPACE	Association for the Care of People with Cerebral Palsy
CIMT	constraint-induced movement therapy
CNS	central nervous system
CP	cerebral palsy
GMFCS	gross motor function classification system
LTPA	physical activity in leisure time
M2M	movement with music
NR	not reported
PA	physical activity
WHO	World Health Organization
SCPE	surveillance of cerebral palsy in Europe

## References

[1] ASPACE ¿Qué es la Parálisis Cerebral? 2025. https://www.aspace.org/que-es.

[2] Manual MSD (2025). Parálisis Cerebral. Manual MSD Versión Para el Público General. https://www.msdmanuals.com/es/hogar/salud-infantil/trastornos-neurol%C3%B3gicos-en-ni%C3%B1os/par%C3%A1lisis-cerebral.

[3] Patel D.R., Bovid K.M., Rausch R., Ergun-Longmire B., Goetting M., Merrick J. (2024). Cerebral palsy in children: A clinical practice review. Curr. Probl. Pediatr. Adolesc. Health Care.

[4] National Institute of Neurological Disorders and Stroke (NINDS) (2025). Cerebral Palsy: Hope Through Research. https://www.ninds.nih.gov.

[5] Salomon I. (2024). Neurobiological insights into cerebral palsy: A review of the mechanisms and therapeutic strategies. Front. Neurol..

[6] Jiang Y., Liu G., Deng B., Li X., Ren J., Zhao Y., Mu X. (2025). White matter lesions and DTI metrics related to various types of dysfunction in cerebral palsy: A meta-analysis and systematic review. PLoS ONE.

[7] Latorre-García J. (2017). Desarrollo de un Programa de Actividad Acuática Como Refuerzo al Tratamiento de Fisioterapia en Bebés con Parálisis Cerebral. Ph.D. Thesis.

[8] McIntyre S., Goldsmith S., Webb A., Ehlinger V., Hollung S.J., McConnell K., Arnaud C., Smithers-Sheedy H., Oskoui M., Khandaker G. (2022). Global prevalence of cerebral palsy: A systematic analysis. Dev. Med. Child Neurol..

[9] Surveillance of Cerebral Palsy in Europe: SCPE (2023). Surveillance of Cerebral Palsy in Europe: About the SCPE Network, a Brief History and Main Achievements. https://scpe.edu.eacd.org/sites/default/files/General_text_about_the_network_history_2023.pdf.

[10] Herrera Sterren N., Fantini F., Berra S. (2024). Therapies, bonds and quality of life of children and adolescents with cerebral palsy: Experiences and perceptions of their caregivers during the pandemic. Andes Pediátrica.

[11] Taylor N.F., Dodd K.J., Larkin H. (2004). Adults with cerebral palsy benefit from participating in a strength training programme at a community gymnasium. Disabil. Rehabil..

[12] Bax M., Goldstein M., Rosenbaum P., Leviton A., Paneth N., Dan B., Jacobsson B., Damiano D. (2005). Proposed definition and classification of cerebral palsy. Dev. Med. Child Neurol..

[13] Verschuren O., Darrah J., Novak I., Ketelaar M., Wiart L. (2014). Health-enhancing physical activity in children with cerebral palsy: More of the same is not enough. Phys. Ther..

[14] Rentinck I.C.M., Ketelaar M., Jongmans M.J., Gorter J.W. (2007). Parents of children with cerebral palsy: A review of factors related to the process of adaptation. Child Care Health Dev..

[15] Bottcher L. (2010). Children with Spastic Cerebral Palsy, Their Cognitive Functioning, and Social Participation: A Review. Child Neuropsychol..

[16] Baron I.S., Kerns K.A., Müller U., Ahronovich M.D., Litman F.R. (2012). Executive functions in extremely low birth weight and late-preterm preschoolers: Effects on working memory and response inhibition. Child Neuropsychol..

[17] Lemay M., Lê T.-T., Lamarre C. (2012). Deficits in two versions of a sustained attention test in adolescents with cerebral palsy. Dev. Neurorehabilit..

[18] Pirila S., van der Meere J., Korhonen P., RuusuNiemi P., Kyntaja M., Nieminen P., Korpela R. (2004). A retrospective neurocognitive study in children with spastic diplegia. Dev. Neuropsychol..

[19] Gutiérrez-Arenas V., Barcelata-Eguiarte B.E., Victoria-Cruz R. (2025). Adaptación y validación del Cuestionario de Calidad de Vida para Niños y Adolescentes con Parálisis Cerebral (CP QOL) en población mexicana. Know Share Psychol..

[20] Vitrikas K., Dalton H., Breish D. (2020). Cerebral palsy: An overview. Am. Fam. Physician.

[21] Paul S., Nahar A., Bhagawati M., Kunwar A.J. (2022). A review on recent advances of cerebral palsy. Oxidative Med. Cell. Longev..

[22] Palisano R.J., Copeland W.P., Galuppi B.E. (2007). Performance of physical activities by adolescents with cerebral palsy. Phys. Ther..

[23] Huroy M., Behlim T., Andersen J., Buckley D., Fehlings D., Kirton A., Pigeon N., Mishaal R.A., Wood E., Shevell M. (2022). Stability of the Gross Motor Function Classification System over time in children with cerebral palsy. Dev. Med. Child Neurol..

[24] Lauruschkus K., Hallström I., Westbom L., Nordmark E. (2015). Participation in physical activities for children with physical disabilities: Feasibility and effectiveness of individualised physical activity referrals. Physiotherapy.

[25] WHO (World Health Organization) (2020). WHO Guidelines on Physical Activity and Sedentary Behaviour. https://www.who.int/publications/i/item/9789240015128.

[26] González-Carbonell I., Brizuela G., Romero-Ávila J.L. (2015). Pedaleo de brazos en personas con lesión medular, parálisis cerebral o ataxia cerebelosa: Parámetros fisiológicos. Rev. Int. De Cienc. Del Deporte.

[27] Hutzler Y., Chacham A., Bergman U., Szeinbver A. (1998). Effects of a movement and swimming program on vital capacity and water orientation skills of children with cerebral palsy. Dev. Med. Child Neurol..

[28] Terada K., Satonaka A., Terada Y., Suzuki N. (2017). Training effects of wheelchair dance on aerobic fitness in bedridden individuals with severe athetospastic cerebral palsy rated to GMFCS level V. Eur. J. Phys. Rehabil. Med..

[29] Van den Berg-Emons R.J., Van Baak M.A., Speth L., Saris W.H. (1998). Physical training of school children with spastic cerebral palsy: Effects on daily activity, fat mass and fitness. Int. J. Rehabil. Res..

[30] Darrah J., Wessel J., Nearingburg P., O’Connor M. (1999). Evaluation of a community fitness program for adolescents with cerebral palsy. Pediatr. Phys. Ther..

[31] Olsen J.E., Ross S.A., Foreman M.H., Engsberg J.R. (2013). Changes in muscle activation following ankle strength training in children with spastic cerebral palsy: An electromyography feasibility case report. Phys. Occup. Ther. Pediatr..

[32] Wu M., Kim J., Arora P., Gaebler-Spira D.J., Zhang Y. (2017). Effects of the integration of dynamic weight shifting training into treadmill training on walking function of children with cerebral palsy: A randomized controlled study. Am. J. Phys. Med. Rehabil..

[33] Martínez-Gómez D., Martínez-de-Haro V., Pozo T., Marcos A., Calle M., Veiga O.L. (2020). Análisis de factores motivacionales en la práctica de actividad física del alumnado de educación secundaria obligatoria en un centro de Valencia. Sci. J. Sch. Sport Phys. Educ. Psychomot..

[34] Satonaka A., Suzuki N. (2018). Aerobic fitness and lifestyle with non-exercise physical activity in adults with cerebral palsy. J. Phys. Fit. Sports Med..

[35] Murphy N.A., Carbone P.S., Council on Children with Disabilities (2008). Promoting the participation of children with disabilities in sports, recreation, and physical activities. Pediatrics.

[36] Page M.J., McKenzie J.E., Bossuyt P.M., Boutron I., Hoffmann T.C., Mulrow C.D., Moher D. (2021). The PRISMA 2020 statement: An updated guideline for reporting systematic reviews. BMJ.

[37] Sánchez-Meca J. (2022). Revisiones Sistemáticas Y Meta-Análisis En Educación: Un Tutorial. RiiTE Rev. Interuniv. Investig. Tecnol. Educ..

[38] Guijarro E., Rocamora I., Evangelio C., González Víllora S. (2020). El modelo de Educación Deportiva en España: Una revisión sistemática. Retos Nuevas Tend. Educ. Física Deporte Recreación.

[39] Chu T.L., Zhang T. (2018). Motivational processes in Sport Education programs among high school students: A systematic review. Eur. Phys. Educ. Rev..

[40] Rodrigues De Sousa Junior R., Oliveira Souto D., Ribeiro Ferreira F., Caetano Martins Da Silva E Dutra F., Resende Camargos A.C., Clutterbuck G.L., de Oliveira J.V.B. (2024). Parents’ perceptions of a modified sports intervention for children with cerebral palsy. Dev. Med. Child Neurol..

[41] Rezavandzayeri F., Suarez H.V., Khortabi A., Carral J.M.C. (2024). The effects of boccia training load on emotional intelligence and quality of life in individuals with cerebral palsy. Retos Nuevas Tend. Educ. Física Deporte Recreación.

[42] Prosser L.A., Pierce S.R., Skorup J.A., Paremski A.C., Alcott M., Bochnak M., Ruwaih N., Jawad A.F. (2024). Motor training for young children with cerebral palsy: A single-blind randomized controlled trial. Dev. Med. Child Neurol..

[43] Ogonowska-Slodownik A., Güeita-Rodriguez J., Skomorowska K., Morgulec-Adamowicz N. (2024). Effects on function and enjoyment of aquatic therapy in children with cerebral palsy: A pilot study in a special education school. Int. J. Disabil. Dev. Educ..

[44] Benito A.G., Anglés V., Périz V.M., Romo L., Artigas E., Mendoza A.J., Programa de Ballet Adaptado en Niños con Hemiplejia (2024). Revista Sanitaria de Investigación. https://revistasanitariadeinvestigacion.com/programa-de-ballet-adaptado-en-ninos-con-hemiplejia/.

[45] Yılmaz D.A., Yildiz M., Yildirim M.S., Ozlenir M. (2023). The effects of core stability exercises on proprioception and balance in children with hemiplegic cerebral palsy. Retos Nuevas Tend. Educ. Física Deporte Recreación.

[46] Wang T.N., Liang K.J., Liu Y.C., Shieh J.Y., Chen H.L. (2023). Effects of intensive versus distributed Constraint-Induced Movement Therapy for children with unilateral cerebral palsy: A quasi-randomized trial. Neurorehabilit. Neural Repair.

[47] Григус І.М., Нагoрна О.Б. (2023). Метoд танцювальнo-рухoвoї терапії дітей, які пoтребують паліативнoї дoпoмoги. Rehabil. Recreat..

[48] Hulst R.Y., Gorter J.W., Obeid J., Voorman J.M., van Rijssen I.M., Gerritsen A., Visser-Meily J.M.A., Pillen S., Verschuren O. (2023). Accelerometer-measured physical activity, sedentary behavior, and sleep in children with cerebral palsy and their adherence to the 24-hour activity guidelines. Dev. Med. Child Neurol..

[49] Hamed S.A., ElMeligie M.M., Kentiba E. (2023). The effects of Halliwick aquatic exercises on gross motor function of children aged from 3 to 5 years with spastic cerebral palsy. Pedagog. Phys. Cult. Sports.

[50] Cribb C.F., Keko M., Creveling S., Rochani H.D., Modlesky C.M., Colquitt G. (2023). Mental health, physical activity, and sports among children with cerebral palsy. Child Care Health Dev..

[51] Towns M., Lindsay S., Arbour-Nicitopoulos K., Mansfield A., Wright F.V. (2022). Balance confidence and physical activity participation of independently ambulatory youth with cerebral palsy: An exploration of youths’ and parents’ perspectives. Disabil. Rehabil..

[52] Lee J., Suk M.H., Yoo S., Kwon J.Y. (2022). Physical Activity Energy Expenditure Predicts Quality of Life in Ambulatory School-Age Children with Cerebral Palsy. J. Clin. Med..

[53] Lai B., Rimmer J., Kim Y., Wen H., Swanson-Kimani E., Davis D. (2022). Home-based Telehealth Movement-to-Music Increases Physical Activity Participation Among Adolescents with Cerebral Palsy: Pilot RCT. Arch. Phys. Med. Rehabil..

[54] Arruda R.C.B.F.D., Tassitano R.M., Brito A.L.D.S., Martins O.S.D.S., Cabral P.C., Antunes M.M.D.C. (2022). Physical activity, sedentary time and nutritional status in Brazilian children with cerebral palsy. J. Pediatr..

[55] Reedman S.E., Boyd R.N., Ziviani J., Elliott C., Ware R.S., Sakzewski L. (2021). Participation predictors for leisure-time physical activity intervention in children with cerebral palsy. Dev. Med. Child Neurol..

[56] Østergaard C.S., Pedersen N.S.A., Thomasen A., Mechlenburg I., Nordbye-Nielsen K. (2021). Pain is frequent in children with cerebral palsy and negatively affects physical activity and participation. Acta Paediatr..

[57] Degerstedt F., Björklund M., Keisu B.I., Enberg B. (2021). Unequal physical activity among children with cerebral palsy in Sweden—A national registry study. Health Sci. Rep..

[58] Williams S.A., McFadden L.M., Blackmore A.M., Davey P., Gibson N. (2020). Do adolescents with cerebral palsy meet recommendations for healthy weight and physical activity behaviours?. Disabil. Rehabil..

[59] Wentz E.E., Bjornson K.F., Kerfeld C.I., Cicirello N., Fiss A.L. (2020). Walking performance, physical activity, and validity of the early activity scale for endurance in young children with cerebral palsy. Phys. Occup. Ther. Pediatr..

[60] Smit D.J., Zwinkels M., Takken T., Hulst R.Y., de Groot J.F., Lankhorst K., Verschuren O. (2020). Sleep quantity and its relation with physical activity in children with cerebral palsy; insights using actigraphy. J. Paediatr. Child Health.

[61] Jami-Vargas P., Caisapanta-Acaro N., Zambrano-Pintado R., Bonilla-Jurado D. (2020). Matrogymnasia and motor development in children between 7 and 8 years old with cerebral palsy. Retos.

[62] Gerber C.N., Carcreff L., Paraschiv-Ionescu A., Armand S., Newman C.J. (2020). Multidimensional measures of physical activity and their association with gross motor capacity in children and adolescents with cerebral palsy. Sensors.

[63] Bjornson K., Fiss A., Avery L., Wentz E., Kerfeld C., Cicirello N., Hanna S.E. (2020). Longitudinal trajectories of physical activity and walking performance by gross motor function classification system level for children with cerebral palsy. Disabil. Rehabil..

[64] Reedman S.E., Boyd R.N., Trost S.G., Elliott C., Sakzewski L. (2019). Efficacy of participation-focused therapy on performance of physical activity participation goals and habitual physical activity in children with cerebral palsy: A randomized controlled trial. Arch. Phys. Med. Rehabil..

[65] Orlando J.M., Pierce S., Mohan M., Skorup J., Paremski A., Bochnak M., Prosser L.A. (2019). Physical activity in non-ambulatory toddlers with cerebral palsy. Res. Dev. Disabil..

[66] Morris A., Imms C., Kerr C., Adair B. (2019). Sustained participation in community-based physical activity by adolescents with cerebral palsy: A qualitative study. Disabil. Rehabil..

[67] Bar-Haim S., Aviram R., Shkedy Rabani A., Amro A., Nammourah I., Al-Jarrah M., Raanan Y., Loeppky J.A., Harries N. (2019). Effects of Exercise Interventions on Habitual Physical Activity and Sedentary Behavior in Adolescents With Cerebral Palsy. Pediatr. Exerc. Sci..

[68] Keawutan P., Bell K.L., Oftedal S., Davies P.S., Ware R.S., Boyd R.N. (2018). Relationship between habitual physical activity, motor capacity, and capability in children with cerebral palsy aged 4–5 years across all functional abilities. Disabil. Health J..

[69] Keawutan P., Bell K.L., Oftedal S., Davies P.S., Ware R.S., Boyd R.N. (2018). Quality of life and habitual physical activity in children with cerebral palsy aged 5 years: A cross-sectional study. Res. Dev. Disabil..

[70] Figueiredo P.R.P., Mancini M.C., Brandão M.D.B. (2018). “Vai jogar?” Fatores que influenciam a participação de adolescentes com paralisia cerebral na educação física escolar. Movimento.

[71] Schasfoort F.C., Dallmeijer A., Pangalila R.F., Catsman C., Stam H.J., Becher J., Bussmann J.B. (2017). Value of botulinum toxin injections preceding a comprehensive rehabilitation period for children with spastic cerebral palsy: A cost-effectiveness study. J. Rehabil. Med..

[72] Keawutan P., Bell K.L., Oftedal S., Davies P.S., Ware R.S., Boyd R.N. (2017). Habitual physical activity in children with cerebral palsy aged 4 to 5 years across all functional abilities. Pediatr. Phys. Ther..

[73] Hahn R.H., Fernández M.C. (2017). ¿Es efectivo el uso combinado de plantillas posturales y órtesis tobillo-pie en la mejora del equilibrio estático y funcional en niños con parálisis cerebral?. Evidentia Rev. Enfermería Basada Evid..

[74] Antón D.M.L. (2017). Efectos de la Hipoterapia en Posición Sedente Lateral Sobre la Espasticidad de los Músculos Aductores de Cadera en Personas con Parálisis Cerebral. Ph.D. Thesis.

[75] Oftedal S., Davies P.S.W., Boyd R.N., Stevenson R.D., Ware R.S., Keawutan P., Benfer K.A., Bell K.L. (2016). Longitudinal Growth, Diet, and Physical Activity in Young Children With Cerebral Palsy. Pediatrics.

[76] Maher C.A., Toohey M., Ferguson M. (2016). Physical activity predicts quality of life and happiness in children and adolescents with cerebral palsy. Disabil. Rehabil..

[77] Bania T.A., Dodd K.J., Baker R.J., Graham H.K., Taylor N.F. (2016). The effects of progressive resistance training on daily physical activity in young people with cerebral palsy: A randomised controlled trial. Disabil. Rehabil..

[78] Aidar F.J., Carneiro A., de Matos D.G., Garrido N.D., dos Santos M.D.M., Aidar L.Z., de Souza R.F., Reis V.M. (2016). Cognitive and functional performance of children with cerebral palsy undergoing physical aquatic activities/Desempenho cognitivo e funcional de crianças com paralisia cerebral submetidas a prática de atividades físicas aquáticas. Motricidade.

[79] Ryan J.M., Hensey O., McLoughlin B., Lyons A., Gormley J. (2015). Associations of sedentary behaviour, physical activity, blood pressure and anthropometric measures with cardiorespiratory fitness in children with cerebral palsy. PLoS ONE.

[80] Mitchell L.E., Ziviani J., Boyd R.N. (2015). Variability in measuring physical activity in children with cerebral palsy. Med. Sci. Sports Exerc..

[81] Mitchell L.E., Ziviani J., Boyd R.N. (2015). Characteristics associated with physical activity among independently ambulant children and adolescents with unilateral cerebral palsy. Developmental Med. Child Neurol..

[82] Balemans A.C., Van Wely L., Becher J.G., Dallmeijer A.J. (2015). Longitudinal Relationship among Physical Fitness, Walking-Related Physical Activity, and Fatigue in Children with Cerebral Palsy. Phys. Ther..

[83] Van Wely L., Balemans A.C., Becher J.G., Dallmeijer A.J. (2014). Physical Activity Stimulation Program for Children with Cerebral Palsy Did Not Improve Physical Activity: A Randomised Trial. J. Physiother..

[84] Van Wely L., Balemans A.C., Becher J.G., Dallmeijer A.J. (2014). The Effectiveness of a Physical Activity Stimulation Programme for Children with Cerebral Palsy on Social Participation, Self-Perception and Quality of Life: A Randomized Controlled Trial. Clin. Rehabil..

[85] Shkedy Rabani A., Harries N., Namoora I., Al-Jarrah M.D., Karniel A., Bar-Haim S. (2014). Duration and Patterns of Habitual Physical Activity in Adolescents and Young Adults with Cerebral Palsy. Dev. Med. Child Neurol..

[86] Lin C.Y., Chang Y.M. (2014). Increase in Physical Activities in Kindergarten Children with Cerebral Palsy by Employing MaKey–MaKey-Based Task Systems. Res. Dev. Disabil..

[87] Bania T.A., Taylor N.F., Baker R.J., Graham H.K., Karimi L., Dodd K.J. (2014). Gross Motor Function Is an Important Predictor of Daily Physical Activity in Young People with Bilateral Spastic Cerebral Palsy. Dev. Med. Child Neurol..

[88] Tang K.T., Richardson A.M., Maxwell D., Spence W.D., Stansfield B.W. (2013). Evaluation of an Activity Monitor for the Objective Measurement of Free-Living Physical Activity in Children with Cerebral Palsy. Arch. Phys. Med. Rehabil..

[89] Song C.S. (2013). Relationships between Physical and Cognitive Functioning and Activities of Daily Living in Children with Cerebral Palsy. J. Phys. Ther. Sci..

[90] Paternina D. (2013). La Hipoterapia: Abordaje Terapéutico de un Caso y sus Logros. Rev. Colomb. Cienc. Anim..

[91] Howcroft J., Klejman S., Fehlings D., Wright V., Zabjek K., Andrysek J., Biddiss E. (2012). Active Video Game Play in Children with Cerebral Palsy: Potential for Physical Activity Promotion and Rehabilitation Therapies. Arch. Phys. Med. Rehabil..

[92] Sandlund M., Lindh Waterworth E., Häger C. (2011). Using Motion Interactive Games to Promote Physical Activity and Enhance Motor Performance in Children with Cerebral Palsy. Dev. Neurorehabilit..

[93] Van Wely L., Becher J.G., Reinders-Messelink H.A., Lindeman E., Verschuren O., Verheijden J., Dallmeijer A.J. (2010). LEARN 2 MOVE 7-12 Years: A Randomized Controlled Trial on the Effects of a Physical Activity Stimulation Program in Children with Cerebral Palsy. BMC Pediatr..

[94] Maher C.A., Williams M.T., Olds T.I.M., Lane A.E. (2010). An Internet-Based Physical Activity Intervention for Adolescents with Cerebral Palsy: A Randomized Controlled Trial. Dev. Med. Child Neurol..

[95] Maltais D.B., Pierrynowski M.R., Galea V.A., Bar-Or O.D.E.D. (2005). Physical Activity Level Is Associated with the O_2_ Cost of Walking in Cerebral Palsy. Med. Sci. Sports Exerc..

[96] Maltais D.B., Pierrynowski M.R., Galea V.A., Matsuzaka A., Bar-Or O. (2005). Habitual Physical Activity Levels Are Associated with Biomechanical Walking Economy in Children with Cerebral Palsy. Am. J. Phys. Med. Rehabil..

[97] Chad K.E., Bailey D.A., McKay H.A., Zello G.A., Snyder R.E. (1999). The Effect of a Weight-Bearing Physical Activity Program on Bone Mineral Content and Estimated Volumetric Density in Children with Spastic Cerebral Palsy. J. Pediatr..

[98] Ganz F., Wright V., Manns P.J., Pritchard L. (2022). Is Physical Activity–Related Self-Efficacy Associated with Moderate to Vigorous Physical Activity and Sedentary Behaviour among Ambulatory Children with Cerebral Palsy?. Physiother. Can..

[99] Roth J., Severtsen B., Hoeksel R., Eddy L. (2022). The Experience of Physical Activity in Adolescents with Cerebral Palsy. Orthop. Nursing.

[100] OMS (1975). Official Records of the World Health Organization.

[101] Wilson M.G., Ellison G.M., Cable N.T. (2016). Basic Science behind the Cardiovascular Benefits of Exercise. Br. J. Sports Med..

[102] Rhodes R.E., Janssen I., Bredin S.S.D., Warburton D.E.R., Bauman A. (2017). Physical Activity: Health Impact, Prevalence, Correlates and Interventions. Psychol. Health.

[103] Anziska Y., Inan S. (2014). Exercise in Neuromuscular Disease. Semin. Neurol..

[104] Gómez L.G. (2018). El Slackline Como Herramienta de Rehabilitación en Niños y Adolescentes con Parálisis Cerebral: Un Ensayo Clínico. Ph.D. Thesis.

[105] Verazaluce-Rodríguez P.R., Rodríguez-Martínez P., Neri-Gámez S., Hernández-Aquino R.M. (2014). Evolución de la Marcha en Pacientes con Parálisis Cerebral y Desplazamiento Asistido, Mediante su Entrenamiento con Equipo de Asistencia Robótica. Rehabilitación.

[106] Cabrera-Martos I., Ortiz-Rubio A., Benitez-Feliponi A., Ramírez M.M., Casilda-López J., Valenza M.C. (2017). Capacidades Físicas y Motoras del Miembro Superior y su Relación con la Independencia Funcional en Parálisis Cerebral Infantil. Fisioterapia.

[107] McKeon M., Slevin E., Taggart L. (2013). A Pilot Survey of Physical Activity in Men with an Intellectual Disability. J. Intellect. Disabil..

[108] Tollerz L.U., Forslund A.H., Olsson R.M., Lidström H., Holmbäck U. (2015). Children with Cerebral Palsy Do Not Achieve Healthy Physical Activity Levels. Acta Paediatr..

[109] Jacques K.D., Dumond N.R., Andrade S.F., Chaves I.P., Toffol W.C. (2010). Effectiveness of the Hydrotherapy in Children with Chronic Encephalopathy No Progressive of the Childhood: A Systematic Review. Fisioter. Mov..

[110] Pérez R. (2016). Principios De Hidroterapia Y Balneoterapia.

[111] Maniu D.A., Maniu E.A., Benga I. (2013). Effects of an Aquatic Therapy Program on Vital Capacity, Quality of Life and Physical Activity Index in Children with Cerebral Palsy. Hum. Vet. Med..

[112] Rimmer J.H., Braddock D., Pitetti K.H. (1996). Research on Physical Activity and Disability: An Emerging National Priority. Med. Sci. Sports Exerc..

[113] Heller T., McCubbin J.A., Drum C., Peterson J. (2011). Physical Activity and Nutrition Health Promotion Interventions: What Is Working for People with Intellectual Disabilities?. Intellect. Dev. Disabil..

[114] Martin J.J. (2013). Benefits and Barriers to Physical Activity for Individuals with Disabilities: A Social-Relational Model of Disability Perspective. Disabil. Rehabil..

[115] Eather N., Wade L., Pankowiak A., Eime R. (2023). The Impact of Sports Participation on Mental Health and Social Outcomes in Adults: A Systematic Review and the ‘Mental Health through Sport’ Conceptual Model. Syst. Rev..

[116] Jaarsma E.A., Geertzen J.H.B., de Jong R., Dijkstra P.U., Dekker R. (2011). Barriers and facilitators of sports in Dutch Paralympic athletes: An explorative study. Scand. J. Med. Sci. Sports.

[117] Erkilic M., Durak S. (2013). Tolerable and inclusive learning spaces: An evaluation of policies and specifications for physical environments that promote inclusion in Turkish primary schools. Int. J. Incl. Educ..

[118] Gamonales J.M. (2016). La educación física como herramienta de inclusión. Rev. Prof. Investig. Docencia Recur. Didácticos.

[119] Hernández-Beltrán V., Gámez-Calvo L., Gamonales J.M. (2020). Propuesta de unidad didáctica para educación física: “Conociendo los deportes para personas con discapacidad visual”. E-Motion Rev. Educ. Mot. Investig..

[120] Gamonales J.M., Campos-Galán S. (2017). Propuesta de unidad didáctica para educación física: Conociendo los deportes paralímpicos. Rev. Prof. Investig. Docencia Recur. Didácticos.

